# Air-quality prediction based on the ARIMA-CNN-LSTM combination model optimized by dung beetle optimizer

**DOI:** 10.1038/s41598-023-36620-4

**Published:** 2023-07-26

**Authors:** Jiahui Duan, Yaping Gong, Jun Luo, Zhiyao Zhao

**Affiliations:** School of Marine Engineer Equipment, Zhejiang Ocean University, Zhoushan, China

**Keywords:** Atmospheric science, Computational science

## Abstract

Air pollution is a serious problem that affects economic development and people’s health, so an efficient and accurate air quality prediction model would help to manage the air pollution problem. In this paper, we build a combined model to accurately predict the AQI based on real AQI data from four cities. First, we use an ARIMA model to fit the linear part of the data and a CNN-LSTM model to fit the non-linear part of the data to avoid the problem of blinding in the CNN-LSTM hyperparameter setting. Then, to avoid the blinding dilemma in the CNN-LSTM hyperparameter setting, we use the Dung Beetle Optimizer algorithm to find the hyperparameters of the CNN-LSTM model, determine the optimal hyperparameters, and check the accuracy of the model. Finally, we compare the proposed model with nine other widely used models. The experimental results show that the model proposed in this paper outperforms the comparison models in terms of root mean square error (RMSE), mean absolute error (MAE) and coefficient of determination (R^2^). The RMSE values for the four cities were 7.594, 14.94, 7.841 and 5.496; the MAE values were 5.285, 10.839, 5.12 and 3.77; and the R^2^ values were 0.989, 0.962, 0.953 and 0.953 respectively.

## Introduction

Due to industrialization, urbanization, and other factors, air pollution has become increasingly prominent. The air quality index (AQI) is an important index reflecting the level of atmospheric pollution^1^, and its size is closely related to the content of various pollutants in the atmosphere. There are six major pollutants affecting air quality: PM2.5, PM_10_, NO_2_, SO_2_, CO, and O_3_. Continuous exposure to air pollution can cause a variety of diseases, such as respiratory, cardiovascular, neurological, etc., and its harm is increasing^2,3^. Air pollution is the product of multiple factors, and its concentration has non-constant and nonlinear characteristics, which brings difficulties to the forecast of atmospheric environmental quality indicators.

Many academics have suggested many prediction models in recent years. The statistics model, machine learning model, and deep learning model can all be generically categorized as these three types of models. A statistical model bases its explanation of cause and effect on assumptions about the distribution of the data and places a high emphasis on parameter inference.

The application of statistical methods in air quality prediction mainly includes the autoregressive (AR) model, the autoregressive integrated moving average (ARIMA) model, the gray model, and the multiple linear regression (MLR) model^4,5^ proposed an algorithm to assess the pollution level of air quality parameters and create a new air quality index based on the fuzzy reasoning system to predict air quality parameters by AR model. Zhang et al.^6^ examines two different approaches to model development, including GAM and traditional linear regression methods. To show the requirement for first-order differencing^7^, proposed an ARIMA model based on the augmented Dickey-Fuller test for PM2.5 annual data. In order to offer accurate forecasts that can accurately capture seasonal and nonlinear properties^8^, created a seasonally nonlinear gray model to account for seasonal fluctuations in the time series of seasonally fluctuating pollution indicators.

Machine learning models rely on large data sets to predict the future, weakening the convergence problem and focusing on model prediction. Mehmood et al.^9^ discusses the transformation of traditional methods into machine learning methods and analyzes emerging trends to identify potentially valuable research directions. Using machine learning models and methods. Varghese and Kumar^10^ developed a machine learning-based empirical model to predict the laminar combustion rate of air pollutants under high pressure and high temperature conditions, using volume fraction as the independent variable. Zhang et al.^11^ uses machine learning models and methods in order to predict how the unpredictability and variability of the indoor mode can cause excessive adjustment or a deficiency of air quality. Rakholia et al.^12^ developed a model that included factors such as weather conditions, urban traffic, air quality data in residential and industrial areas, urban spatial information, time-series composition, and pollution concentrations. Gu et al.^13^ proposes a new hybrid interpretable prediction machine learning model for PM2.5 prediction that can outperform other models in terms of peak prediction accuracy and model interpretability. Maltare and Vahora^14^ focuses on support vector machine algorithms based on RBF kernel models. Munir^15^ used machine learning models to assess the impact of intelligent transport interventions on air quality.

Deep learning model is more adaptive and easily transformable than a machine learning model, allowing easier adaptation to different domains and applications. Zhang et al.^16^ made a comprehensive review of the Deep Learning Method dedicated to air pollution concentration forecasting. Wu et al.^17^ proposes a hybrid deep learning-based model to predict the pollutant concentration in the next hour at the network scale based on the identified spatio-temporal features. Jurado^18^ developed a fast and accurate system using convolutional networks for real-time forecasting of air pollution based on wind speed, traffic flow, and building geometry. Zhang et al.^19^ gives a meta-learning algorithm for knowledge transfer between cities with large differences that can incorporate spatio-temporal correlations between monitoring stations and transfer data from other cities with rich training data. Saez and Barceló^20^ proposed a new model that can predict the space for long-term and short-term exposure to air pollutants and has relatively low monitoring STA pollutants and a lower calculation time.

Some scholars have developed combinatorial models to improve the accuracy of prediction in pursuit of greater prediction rates. Due to the combination of the advantages of different models, the combined model’s accuracy has greatly improved in prediction accuracy. Kshirsagar^21^ explores the role that neural networks, regression, and hybrid models play in the analysis, prediction, and mitigation of air pollution, taking into account the most recent developments and new research in the field. Zhang and Li^22^ combined the efficient features of CNN with the algorithmic advantages of LSTM to propose a CNN-LSTM model to predict future air pollution data. Vlachokostas^23^ confirmed the regression model by using multiple stepwise regression analysis to find a significant statistical relationship between C_6_H_6_ and CO. Gunasekar et al.^24^ developed a new hybrid model for air quality prediction, optimizing the residual error of ARIMA by the LSTM algorithm. Wang et al.^25^ added an attention mechanism to the model to improve the prediction accuracy of the LSTM model. Dai et al.^26^ established five haze hazard risk assessment models by improving theparticle swarm optimization (IPSO) light gradient boosting machine (LightGBM) algorithm and a hybrid model combining XGBoost, four GARCH models and MLP model (XGBoost-GARCH-MLP) is proposed to predict PM2.5 concentration values and volatility^27^.

With the rapid development of soft computing technologies, many meta-heuristic algorithms have recently been designed and used as competitive alternative solutions to address improved accuracy of predictive models due to their simplicity and ease of implementation.Grey Wolf Optimizer (GWO) is a nature-inspired optimisation algorithm inspired by the behaviour of grey wolves in packs. Its flexibility and efficiency make it a popular optimisation algorithm. Akilandeswari et al.^28^ used LSTM with the Weighted Grey Wolf Optimizer (LSTM-WGWO) to increase the accuracy of the air quality index significantly.The Harris-hawks optimisation algorithm is a nature-inspired group intelligence based optimisation algorithm where the objective is to minimise or maximise an objective function given a constraint. Du et al.^29^ proposes a new multi-objective optimisation version of HHO and develops a new hybrid model to improve the accuracy of the predictive model. PSO is a population intelligence based optimisation algorithm inspired by the behaviour of groups of organisms searching for optimal solutions in the solution space. Huang et al.^30^ improved the PSO algorithm accordingly, optimized the overall prediction performance of BP neural network, adjusted the change strategy of the inertia weight as well as the learning factor, and ensured the diversity of particles during the early stage and the fast convergence to the global optimal solution.The Cuckoo optimisation algorithm is an optimisation algorithm based on the idea of parasitism in a bird’s nest, simulating the biology of a male bird occupying a nest and a hetero bird making the same random behaviour and thus searching for the optimal solution. Sun et al^31^ proposes a hybrid model for cuckoo search optimisation based on principal component analysis (PCA) and least squares support vector machine (LSSVM). The model outperforms a single LSSVM model with default parameters and a general regression neural network (GRNN) model for PM2.5 concentration prediction.In summary, machine learning and deep learning models can handle time series forecasting more accurately than traditional statistical models. However, due to the non-stationary nature of AQI data, it may be difficult for individual models to fully explore the internal regularities among the data. Most of the comparative models chosen by previous models are based on derivatives of the proposed model, do not provide a comprehensive comparison of other models, and have limited accuracy. A new combined model is therefore proposed in this paper. To verify the superiority of the model, the AQI is used as an example for forecasting and four different cities in China are selected for the study to compare the forecasting effectiveness of other models. Beijing, Lanzhou, Jiaozuo and Guangzhou were chosen for the study.

The main contributions of this paper are as follows: (1) The linear part of the data is extracted and fitted using the ARIMA model to output the prediction results of the linear part and the non-linear part, and the output non-linear part is imported into the deep learning model for fitting to obtain the prediction values of the non-linear part. (2) The prediction results of the linear part and the non-linear part are combined to obtain the final prediction output. (3) To avoid the problem of blindness in CNN-LSTM hyperparameter setting, this paper uses a dung beetle optimization algorithm to search for hyperparameters of the CNN-LSTM model.

## Materials and methods

### Statistical method

The ARIMA model is called the average model of the Returning Integration Movement, which is usually written as ARIMA(p, d, q). This model is able to handle non-stationary series and is widely used in algorithmic prediction and has high accuracy in air quality prediction. In the ARIMA model, AR is the autoregressive and p is the number of autoregressive terms; I is the difference and d is the number of differences (order) made to make it a smooth series; MA is the sliding average and q is the number of sliding average terms, and the mathematical model can be represented by (1). In general, second-order differences are single-integer smooth data, i.e., only the ARMA(p, 2, q) model is required. ARMA(p, 2, q) can be transformed into AR() and MA(), which correspond to the characteristic that both functions exhibit a gradual decay. The p’s and q’s are determined by either the deficit pool information criterion (AIC) or the Bayesian information criterion (BIC). In this paper, BIC is used to determine the values of p and q. The BIC formula is (2).

1 $$\begin{array}{*{20}c} {y_{t} = c + \phi_{1} *y_{t - 1} + \cdots + \phi_{p} *y_{t - p} + \theta_{1} *e_{t - 1} + \cdots \theta_{q} *e_{t - q} } \\ \end{array}$$

2 $$\begin{array}{*{20}c} {B_{BIC} = k*\ln \left( n \right) - 2\ln \left( L \right)} \\ \end{array}$$

where ${y}_{t}$ is the number of difference levels, $c$ is a constant value, $\phi$ is the AR parameter (autocorrelation size), $p$ is the number of lags (AR order), $\theta$ is the MA parameter value (error autocorrelation), $q$ denotes the number of lags (order of the model MA), and ${e}_{t}$ is the error^32^. $k$ is the number of model parameters, $n$ is the number of samples, and $L$ is the likelihood function.

### Machine learning model

#### RF

Random forest is an integrated prediction model based on decision trees, which integrates multiple decision trees, each of which has a certain dependence on the independently sampled random vector values, and all decision trees in the random forest have the same distribution. The two most important parameters of RF are Number of trees and Number of features. Number of decision trees indicates the number of trees in the forest andNumber of features indicates the number of randomly selected features for each decision tree^33^).

#### SVM

**Figure 1:**
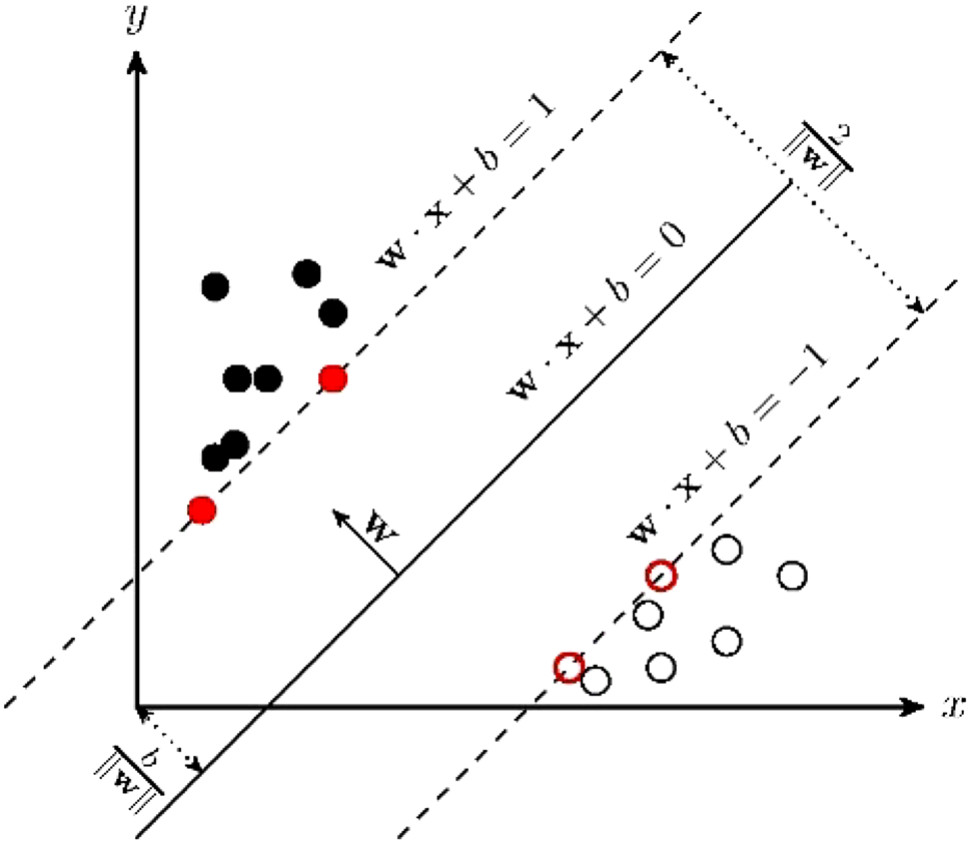
SVM schematic.

A supervised learning algorithm, Support Vector Machine (SVM), is a generalized linear classifier that performs binary classification of data in a supervised learning manner, with its decision boundary being the maximum margin hyperplane of the learned example solution. For example, $\omega \cdot x+b=0$ is separation hyperplane in Fig. 1.

The SVM model parameters include: kernel function, penalty coefficient, regularization parameter and accuracy. There are five kernel functions: linear, poly, rbf, sigmoid and pre-computed. This paper choose linear kernel function, mathematical formula for (3); maximum number of iterations Number of iterations of the algorithm.

3 $$\begin{array}{*{20}c} {K\left( {x,z} \right) = x \cdot z} \\ \end{array}$$

### Deep learning model

#### LSTM

**Figure 2:**
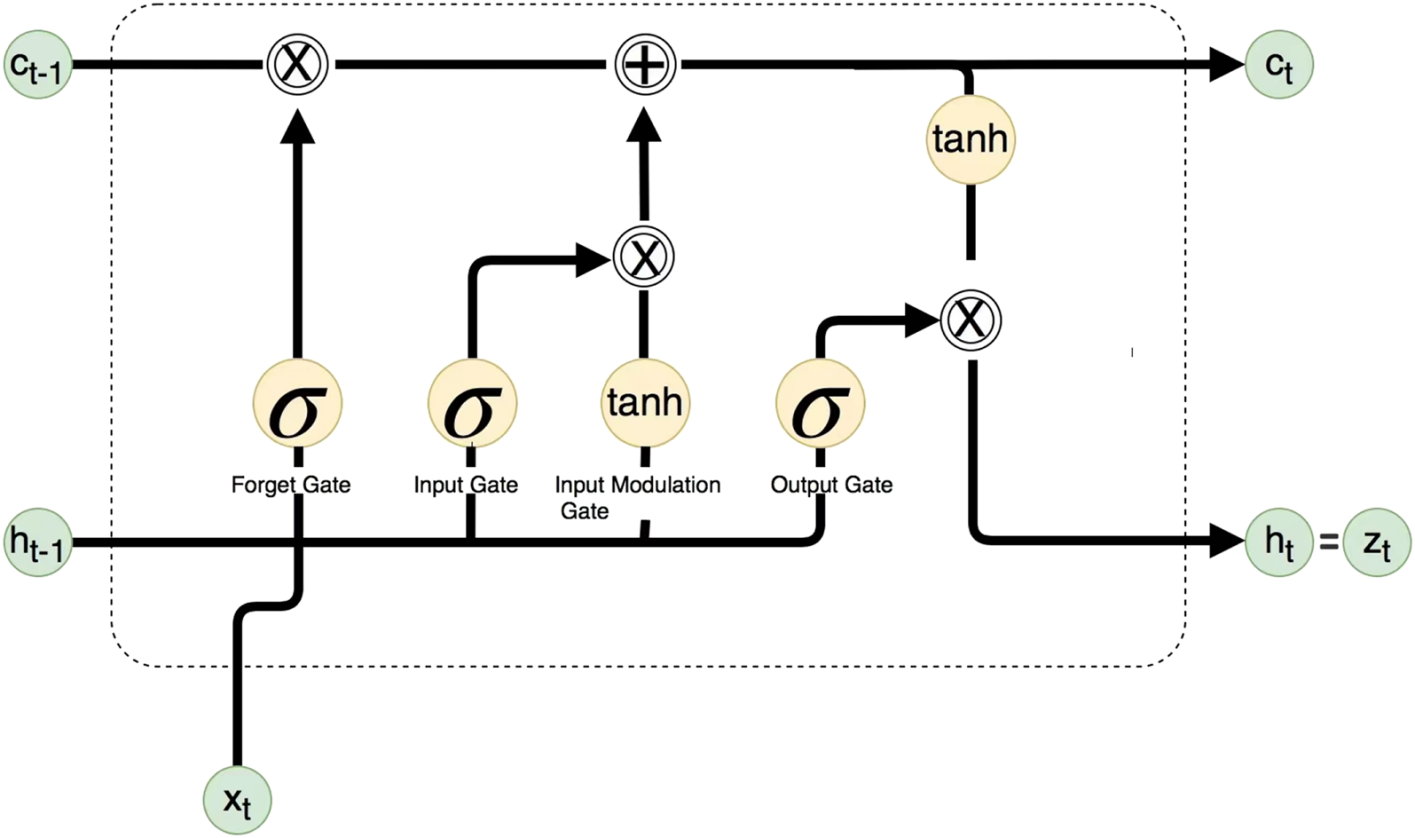
LSTM Structure.

Long-short-term memory (LSTM), as a unique class of recurrent neural networks, is used to solve the gradient diffusion problem in recurrent neural networks. LSTM model contains three main gates, namely: forget gate, memory gate and output gate, and its structure is shown in Fig. 2.

The task of the forget gate is to accept a long-term memory ${C}_{t-1}$ (the output from the previous unit module) and decide which part of ${C}_{t-1}$ to retain and forget, with the mathematical expression (4);

4 $$\begin{array}{*{20}c} {f_{t} = \sigma \left( {W_{f} \cdot \left[ {h_{t - 1} \cdot x_{t} } \right] + b_{f} } \right)} \\ \end{array}$$

The memory gate will forget the attribute information discarded by the gate, locate the corresponding new attribute information in the unit module, and supplement the discarded attribute information. The memory gate is made up of two layers: the sigmoid layer and the tanh layer, which have the mathematical expressions (5), (6), and (7).

5 $$\begin{array}{*{20}c} {i_{t} = \sigma \left( {W_{i} \left[ {h_{t - 1} ,x_{t} } \right] + b_{i} } \right)} \\ \end{array}$$

6 $$\begin{array}{*{20}c} {\tilde{C}_{t} = \tanh \left( {W_{c} \left[ {h_{t - 1} ,x_{t} } \right] + b_{c} } \right)} \\ \end{array}$$

7 $$\begin{array}{*{20}c} {C_{t} = f_{t} \times C_{t - 1} + i_{t} \times \tilde{C}_{t} } \\ \end{array}$$

The output gate is used to determine the cell state output part, and the cell state is processed through the tanh layer, and the two are multiplied to get the final information we want to output, with the mathematical expressions (8) and (9).

8 $$\begin{array}{*{20}c} {o_{t} = \sigma \left( {W_{o} \left[ {h_{t - 1} ,x_{t} } \right] + b_{o} } \right)} \\ \end{array}$$

9 $$\begin{array}{*{20}c} {h_{t} = o_{t} \tanh \left( {C_{t} } \right)} \\ \end{array}$$

where ${h}_{(t-1)}$ represents the previous cell output, ${x}_{t}$ represents the current cell input, $\sigma$ denotes the sigmoid activation function, ${W}_{f}$ represents the forgetting gate’s weight coefficient matrix, and ${b}_{f}$ represents the forget gate bias vector. ${W}_{i}$, ${b}_{i}$ denote the input gate weight coefficient matrix and bias vector determined by the sigmoid activation function, while ${W}_{c}$ and ${b}_{c}$ denote the input gate weight coefficient matrix and bias vector determined by the hyperbolic tangent activation function, respectively. $tanh$ denotes the hyperbolic tangent activation function. ${W}_{o}$, ${b}_{o}$, denote the output gate weight coefficient matrix and bias vector, respectively, and ${o}_{t}$ denotes the output gate at time $t$^34^.

#### DBO

DBO (Dung Beetle Optimizer, DBO for short) is a new population intelligence algorithm based on beetle ball rolling, dancing, foraging, stealing, reproduction and other behaviors. This algorithm is characterized by strong merit-seeking ability and fast convergence. The DBO algorithm consists of four main processes: ball rolling, breeding, foraging and stealing. In the case of dung beetle unobstructed ball rolling, assuming that light intensity affects dung beetle position, the formula for updating the dung beetle’s position as follows.${x}_{i} (t+1)={x}_{i} (t)+\alpha \cdot k\cdot {x}_{i} (t-1)+b\cdot \Delta x$

10 $$\begin{array}{*{20}c} {\Delta x = \left| {x_{i} \left( t \right) - X^{w} } \right|} \\ \end{array}$$

where $t$ is the current number of iterations, ${x}_{i}\left(t\right)$ is the position information of the $i$ th praying mantis in the $i$ th iteration, and $k\in (\mathrm{0,0.2}]$ is the current number of iterations represents the deflection coefficient’s constant value, $b$ represents the value of the constant assigned to (0, 1), and $\alpha$ represents the natural coefficient assigned to − 1 or 1. ${X}^{w}$ represents the ball’s worst position, and $\Delta x$ is used to simulate the change in light intensity^35^.

When the dung beetle encounters an obstacle that prevents it from progressing, it adjusts by dancing to find a new path. The algorithm uses a tangent function to model the dancing behavior. The dung beetle’s position is updated as follows after determining a new direction and continuing to roll the ball.

11 $$x_{i} \left( {t + 1} \right) = x_{i} \left( t \right) + tan\left( \theta \right)\left| {x_{i} \left( t \right) - x_{i} \left( {t - 1} \right)} \right|$$

where $\theta$ is an angle tilted from the $\left[0,\pi \right]$ direction^35^.

In the reproductive process, the scarab algorithm adopts an edge selection strategy to simulate the spawning area of scarabs as Equation.

12 $$\left\{ {\begin{array}{*{20}l} {Lb^{*} = \max \left( {X^{*} \cdot \left( {1 - R} \right),Lb} \right)} \hfill \\ {Ub^{*} = min\left( {X^{*} \cdot \left( {1 - R} \right),Ub} \right)} \hfill \\ \end{array} } \right.$$

where ${X}^{*}$ represents the current optimal solution, while $L{b}^{*}$ represents the optimal solution of the optimal solution, and $U{b}^{*}$ represents the optimal solution of the optimal solution. $R=1-\frac{t}{T}$ and $T$ is the maximum number of iterations, $Lb$ is the upper and lower limits of the optimal solution and $Ub$ is the upper limit of the optimal solution^35^.

When the egg-laying zone is determined, the dung beetle lays only one egg per iteration. It is clear from (12) that the egg-laying area is dynamically adjusted in the iteration and therefore the location of l eggs is also dynamic as Equation^35^.

13 $$\begin{array}{*{20}c} {B_{i} \left( {t - 1} \right) = X^{*} + b_{1} \cdot \left( {B_{i} \left( t \right) - Lb^{*} } \right) + b_{2} \cdot \left( {B_{i} \left( t \right) - Ub^{*} } \right)} \\ \end{array}$$

where, ${B}_{i}\left(t\right)$ is the position of the $i$ th sphere at the $t$ th iteration, ${b}_{1}$ and ${b}_{2}$ are two independent random vectors of size $1\times D$, and $D$ is the dimension of the optimal solution^35^.

During the predation process, the boundary of the optimal predation area is determined according to the position changes of the insects during the predation process.

14 $$\begin{array}{*{20}c} {\left\{ {\begin{array}{*{20}l} {Lb^{b} = \max \left( {X^{b} \cdot \left( {1 - R} \right),Lb} \right)} \hfill \\ {Ub^{b} = \min \left( {X^{b} \cdot \left( {1 + R} \right),Ub} \right)} \hfill \\ \end{array} } \right.} \\ \end{array}$$

where ${X}^{b}$ is the global optimization, $L{b}^{b}$ is the lower bound of the optimal search domain, and $U{b}^{b}$ is the upper bound of the optimal search domain. The location of the little beetle is updated as follows.

15 $$\begin{array}{*{20}c} {x_{i} \left( {t + 1} \right) = x_{i} \left( t \right) + C_{1} \cdot \left( {x_{i} \left( t \right) - Lb^{b} } \right) + C_{2} \cdot \left( {x_{i} \left( t \right) - Ub^{b} } \right)} \\ \end{array}$$

where, ${x}_{i}\left(t\right)$ denotes the position information of the $i$th dung beetle at the $t$th iteration, ${C}_{1}$ denotes a random number obeying normal distribution, and ${C}_{2}$ denotes a random vector belonging to (0, 1)^35^.

During the stealing phase, the location of the thieving dung beetle is updated as follows.

16 $$x_{i} \left( {t - 1} \right) = X^{b} + S \cdot g \cdot \left( {\left| {x_{i} \left( t \right) - X^{*} } \right| + \left| {x_{i} \left( t \right) - X^{b} } \right|} \right)$$

where ${x}_{i}\left(t\right)$ represents the location information of the $i$th thief at the $t$th iteration, $g$ represents a $1\times D$ random vector that obeys a normal distribution, and $S$ represents a constant value^35^.

### Combination model

#### CEEMDAN-CNN-LSTM and CEEMDAN-LSTM

CEEMDAN is an improved algorithm based on EMD. CEEMDAN improves the reconstructed signal by adding a limited amount of white noise consistent with the standard normal distribution in each iteration^36^. The CEEMDAN algorithm solves the EMD sub-modal mixing problem and the EEMD and CEEMD residual white noise problem. The algorithm steps are divided into 4 main steps.

The first step is to introduce Gaussian white noise into the known signal $y\left(t\right)$ to obtain a new signal $y\left(t\right)+{\left(-1\right)}^{q}\varepsilon {\upsilon }^{j}\left(t\right)$, where q = 1 or 2. EMD decomposition is performed on the new signal to generate a characteristic mode component ${C}_{1}$ in the form of Eq. (17). As shown in, the ensemble average of the N mode components generated in the second step yields the first characteristic mode component of the CEEMDAN decomposition (18). The third step, by Eq. (19), calculates the residual after removing the 1st mode component. The preceding process is repeated in the fourth step until the obtained residual signal is a monotonous function. In this way, the number of eigenmodes can be obtained. The original signal y(t) is then decomposed into (20).

17 $$E\left( {y\left( t \right) + \left( { - 1} \right)^{q} \varepsilon \upsilon^{j} \left( t \right)} \right) = C_{1}^{j} \left( t \right) + r^{j}$$

18 $$\overline{{C_{1} \left( t \right)}} = \frac{1}{N}\mathop \sum \limits_{j = 1}^{N} C_{1}^{j} \left( t \right)$$

19 $$r_{1} \left( t \right) = y\left( t \right) - \overline{{C_{1} \left( t \right)}}$$

20 $$y\left( t \right) = \mathop \sum \limits_{k = 1}^{k} \overline{{C_{k} \left( t \right)}} + r_{k} \left( t \right)$$

where ${E}_{i}\left(\cdot \right)$ is the $i$ th eigenmode obtained by EMD decomposition, $\overline{{C}_{i}\left(t\right)}$ is the $i$th eigenmode obtained by CEEMDAN decomposition, ${\upsilon }^{j}$ is composed of Gaussian noise (Gaussian noise) , $j$ is the amount of white noise added, $\varepsilon$ is the white noise standard table, $y\left(t\right)$ is the decomposed signal.

**Figure 3:**
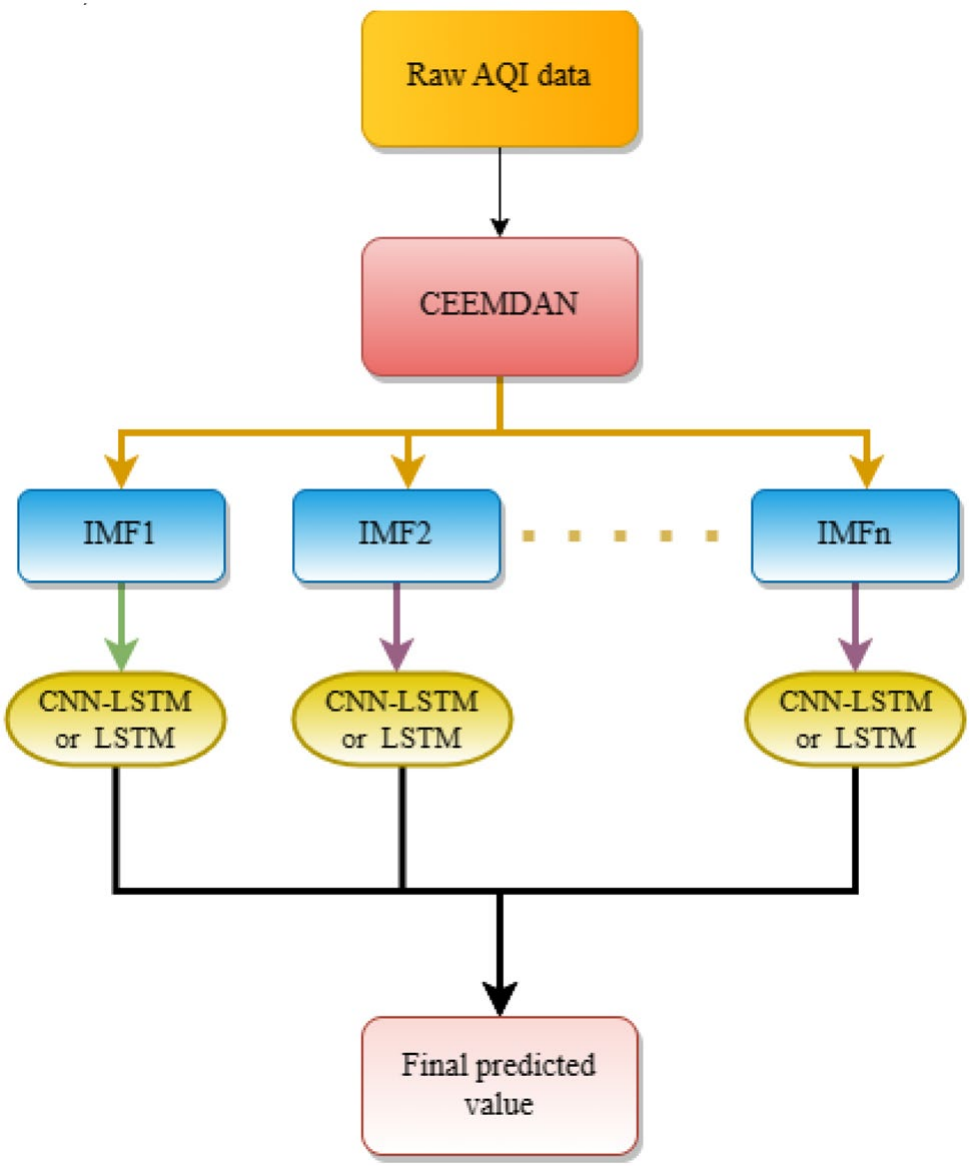
Data decomposition model process.

CEEMDAN-CNN-LSTM and CEEMDAN-LSTM i.e., the CNN-LSTM model is used to fit each IMF component with the LSTM model, and the final prediction is obtained after combining all components, and the process is shown in Fig. 3.

#### ARIMA-CNN-LSTM、ARIMA-DBO-LSTM and ARIMA-DBO-CNN-LSTM

Consider the time series data ${x}_{t}$ as the combination of linear component ${L}_{t}$ and nonlinear component ${N}_{t}$ represented by (21). Since linear and nonlinear modeling methods have their own characteristics, the former can only identify linear features of time series, while the latter can effectively mine them^37^. The ARIMA model can predict short-period linear trends well, while the LSTM model can predict complex, non-linear time series well^32^.

**Figure 4:**
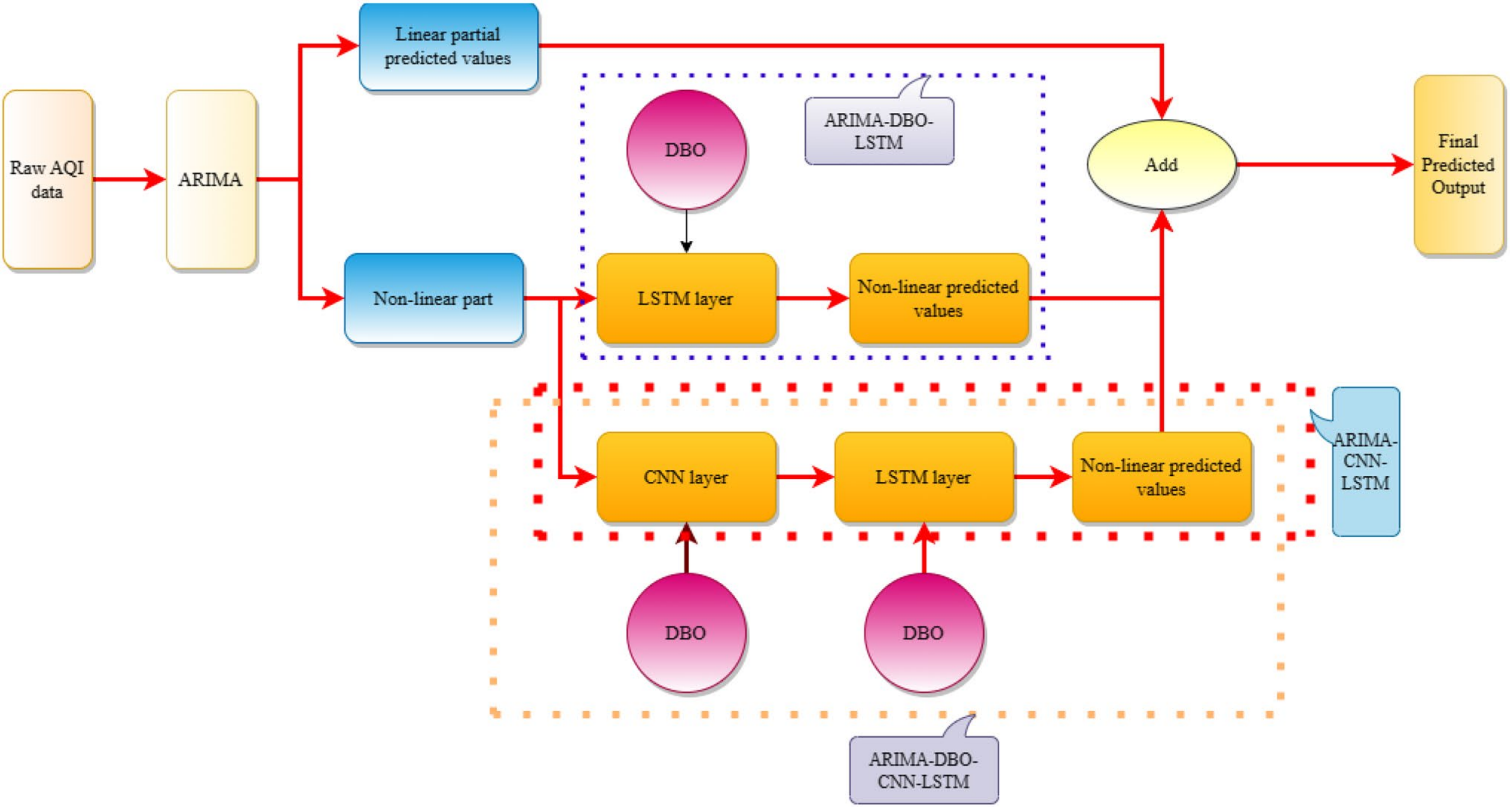
ARIMA-DBO-CNN-LSTM model and derived model process.

The ARIMA model is used to predict the linear and nonlinear components of the data, which is then fed into the deep neural network and fit to obtain the predicted value of the nonlinear component. On this basis, the data of both linear and nonlinear aspects are integrated, and the final prediction result is obtained. In order to overcome the blindness of hyperparameter setting, the dung beetle optimization algorithm is introduced to determine the optimal value of hyperparameter setting, the model flow is shown in Fig. 4.

21 $$x_{t} = L_{t} + N_{t}$$

## Air pollutant concentration prediction

### Study area selection

To verify the prediction effect of the model, AQI data of Beijing, Lanzhou, Jiaozuo and Guangzhou cities in China are selected for the study. Because all four cities are industrial, a large number of industrial emissions cause severe air pollution. The Chinese government has worked to reduce urban air pollution in recent years, but China’s air quality ranking remains at the bottom. Therefore, the air forecasts for these three cities are very important.

**Figure 5:**
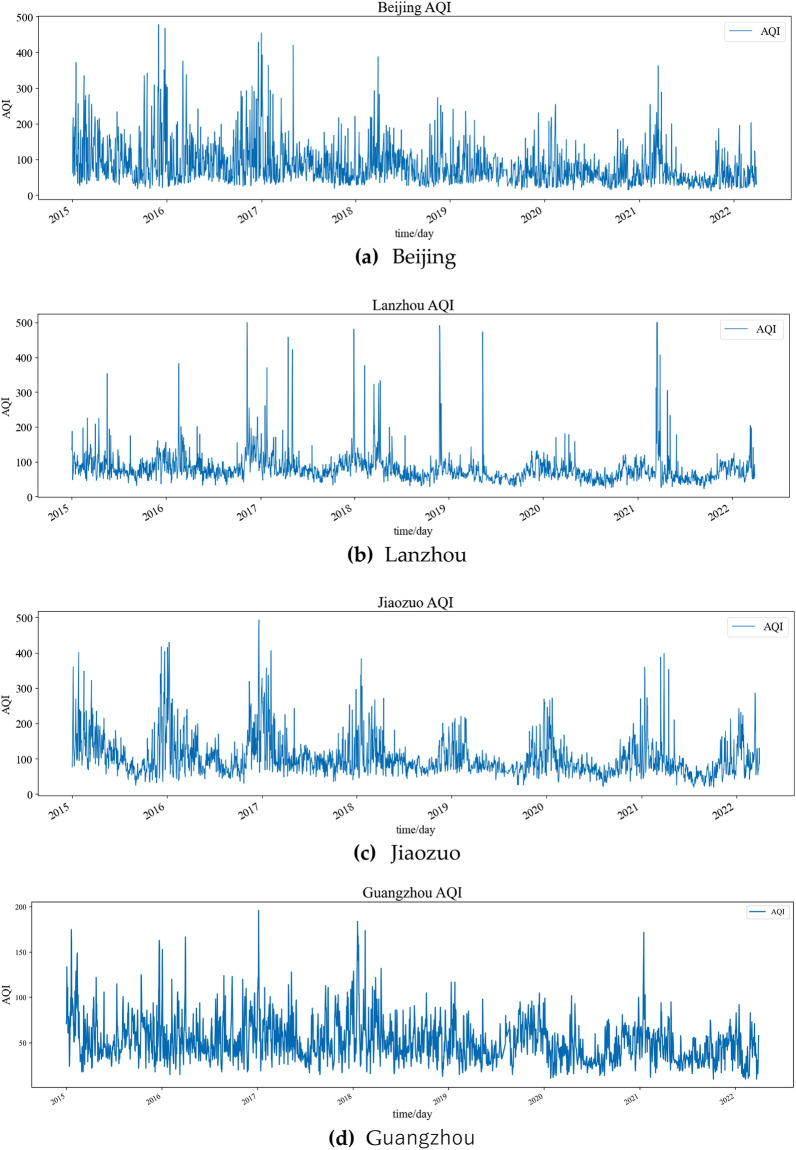
Raw data.

The AQI data for the four cities in this paper were gained by the Resource and Environment Science and Data Center of the Chinese Academy of Sciences (https://www.resdc.cn/Default.aspx), and the data are daily AQI data from January 2015 to March 1, 2022, as shown in Fig. 5.

### Data processing

On this basis, the training samples are divided into two parts, one part accounts for 80% of the training samples, and the other part accounts for 20% of the test samples. Also, in order to improve the training speed of the model, the data are mapped between (0, 1] by a normalization operation. with the normalization formula as follows.

22 $$x_{i} = \frac{{x - x_{min} }}{{xmin_{max} }}$$

After model training and prediction, the data need to be subjected to an inverse normalization operation to facilitate the calculation of the evaluation function and plotting, with the inverse normalization equation being as follows.

23 $$x = \left( {xmin_{max} x_{i} + x_{min} } \right)$$

Among them, ${x}_{i}$ represents the standardized data, ${x}_{max}$ represents the largest data in the array, and ${x}_{min}$ represents the smallest data in the array. This paper chooses three evaluation indicators: root mean square error RMSE, coefficient of determination R^2^, and mean absolute error MAE, and provides specific calculation formulas to accurately compare the prediction effects of each model.

24 $$RMSE = \sqrt {\frac{1}{n}\mathop \sum \limits_{i = 1}^{n} \left( {\overset{\lower0.5em\hbox{$\smash{\scriptscriptstyle\frown}$}}{y}_{i} - y_{i} } \right)^{2} }$$

25 $$R^{2} = \frac{{\mathop \sum \nolimits_{i = 1}^{n} \left( {y_{i} - \overset{\lower0.5em\hbox{$\smash{\scriptscriptstyle\frown}$}}{y}_{i} } \right)^{2} }}{{\mathop \sum \nolimits_{i = 1}^{n} \left( {y_{i} - \overset{\lower0.5em\hbox{$\smash{\scriptscriptstyle\frown}$}}{y} } \right)^{2} }}$$

26 $$MAE = \frac{1}{n}\mathop \sum \limits_{i = 1}^{n} \left| {\overset{\lower0.5em\hbox{$\smash{\scriptscriptstyle\frown}$}}{y}_{i} - y_{i} } \right|$$

where $$n$$ is the sample capacity, $${y}_{i}$$ is the sample value, $$\overline{y}$$ is the mean value, and $$\overset{\lower0.5em\hbox{$\smash{\scriptscriptstyle\frown}$}}{y}_{i}$$ is the predicted value.

### model prediction results

#### Model parameter setting

For the advantages of the ARIMA-DBO-CNN-LSTM model, we compared the classic machine learning model, deep learning model and statistical model, respectively, and the selection of the combined model is not limited to the derivatives of the model proposed in this paper, but also selects the data decomposition combined model which is very widely used at present. And one-dimensional regression equation is used, and multiple experiments are carried out on each equation to ensure that it has the best forecasting effect. On this basis, the maximum constraints on p and q are made using the BIC criterion, and the statistical model restricted the maximum value of p and q to 5. Table 1 shows all parameters in the four cities. The hyperparameter settings of other models are shown in Table 2.

**Table 1:** Statistical model parameter setting.

City	Beijing	Lanzhou	Jiaozuo	Guangzhou
ARIMA parameter setting	(3, 1, 1)	(2, 1, 2)	(3, 1, 1)	(2, 1, 2)

**Table 2:** Model parameter settings.

Model type	Models	Parameter setting
Traditional machine learning models	SVM	kernel = ‘linear’, Other parameters select default
Deep learning models	LSTM	neurons1 = 50, neurons2 = 100, neurons3 = 150, batch_size = 64, epochs = 100, Learning Rate = 0.1, Sliding Window = 10
Combination model	ARIMA-CNN-LSTM	filters = 512, kernel_size = 2, strides = 1, 3 layers of neurons = 50, batch_size = 64, epochs = 100, Learning Rate = 0.2, Sliding Window = 10
	CEEMDAN-CNN-LSTM	filters = 512, kernel_size = 2, strides = 1, neurons = 128, batch_size = 100, epochs = 100, Learning Rate = 0.2, Sliding Window = 10
	CEEMDAN-LSTM	neurons1 = 128, neurons2 = 100, epochs = 100, Learning Rate = 0.2, Sliding Window = 10
Optimization algorithm model		3 layers of neurons = [1,300], Sliding Window = [1,50], Learning Rate = [0.001,0.99], batch_size = [1,300], filters = [1,600], kernel_size = [1,10], strides = [1,5],

#### Forecast comparison and analysis

**Figure 6:**
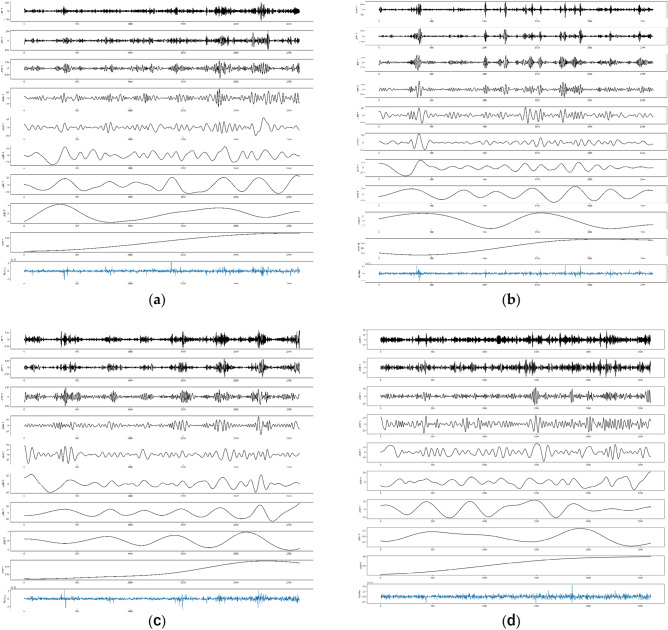
CEEMDAN decomposition.

All models were predicted after the parameters were set, and the CEEMDAN-CNN-LSTM and CEEMDAN-LSTM models AQI data were decomposed by CEEMDAN to obtain 9 or 10 IMF components and one residual component, as shown in Fig. 6.

## Results and analysis

**Figure 7:**
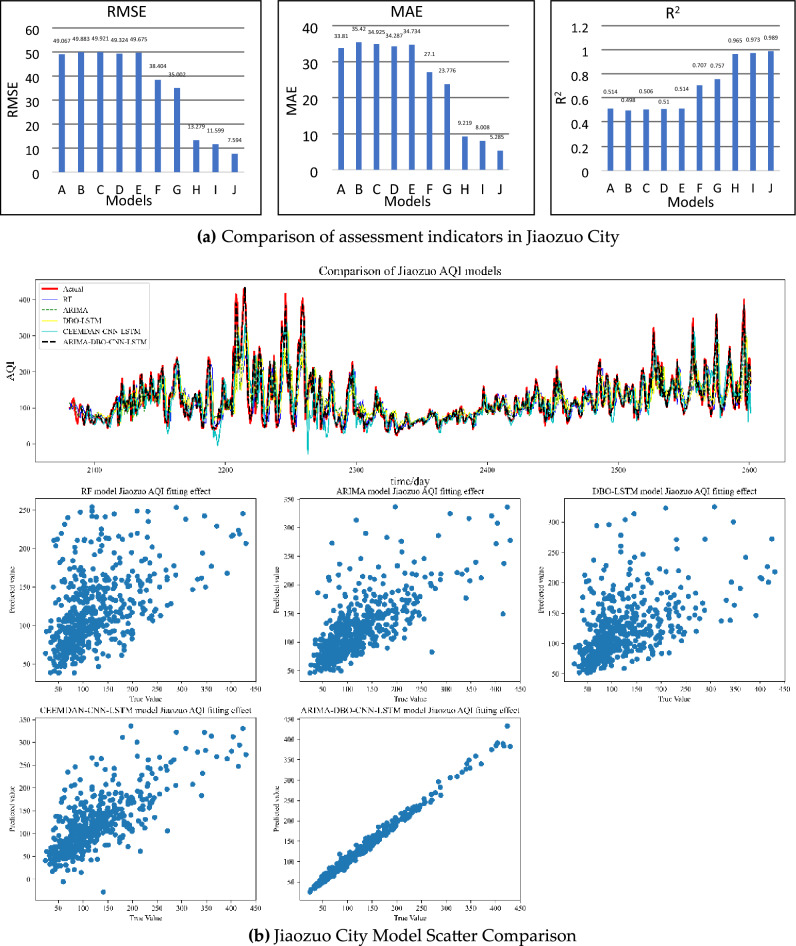
Jiaozuo City Forecast Comparison.

Taking Jiaozuo City as an example, the evaluation indicators obtained after import-ing the evaluation function were compared with the output results of all the models after they had been run. In order to compare the performance of the evaluation metrics of dif-ferent models more intuitively, the evaluation metrics of each model were plotted as bar charts as shown in Fig. 7a. In order to clearly compare the ARIMA-DBO-CNN-LSTM model with other kinds of models, the best-performing model among the models was selected to plot line and scatter plots, as shown in Fig. 7

Through the predicted effect of Jiaozuo City we can easily find:

A. The single model has poor forecasting ability, while the combined model has significantly better forecasting accuracy than the single model.
B. The four types of single prediction models performed similarly, with the R^2^ metric showing a prediction accuracy of around 0.5, with SVM being the worst performer among the traditional machine learning models.
C. By splitting the data, the model’s prediction accuracy can be significantly im-proved. CEEMDAN-RMSE, LSTM’s MAE, and R^2^ decreased by 23.07%, 22.41%, and 39.72%, respectively, when compared to LSTM.
D. The ARIMA-DBO-CNN-LSTM model has a 64.02% reduction in RMSE, 77.78% reduction in MAE and 30.65% improvement in R^2^ relative to the combined data processing model CEEMDAN-CNN-LSTM; and 84.71% reduction in RMSE, 84.78% reduction in MAE and 92.41% improvement in R^2^ relative to the deep learning model DBO-LSTM. 84.78% and an increase in R^2^ of 92.41%.
E. The DBO can effectively improve the prediction accuracy of the model, comparing the ARIMA-DBO-CNN-LSTM model with the ARIMA-CNN-LSTM model, the RMSE metric is reduced by 34.53%, MAE is reduced by 34% and R^2^ is improved by 1.64%.
F. Using different models to predict different parts of the data can effectively im-prove the prediction accuracy and has better results than CEEMDAN decom-posed data. From the comparison between the derivative model of ARI-MA-DBO-CNN-LSTM model and the decomposed combined model, the deriva-tive model can reach above 0.95 in R^2^ index, while the data decomposed com-bined model maintains between 0.7 and 0.8.
G. As can be seen in Fig. 7b, the ARIMA-DBO-CNN-LSTM model can predict the AQI of Jiaozuo City well in comparison with the single model and the combined model, and the scatter plot has the best aggregation effect.

**Figure 8:**
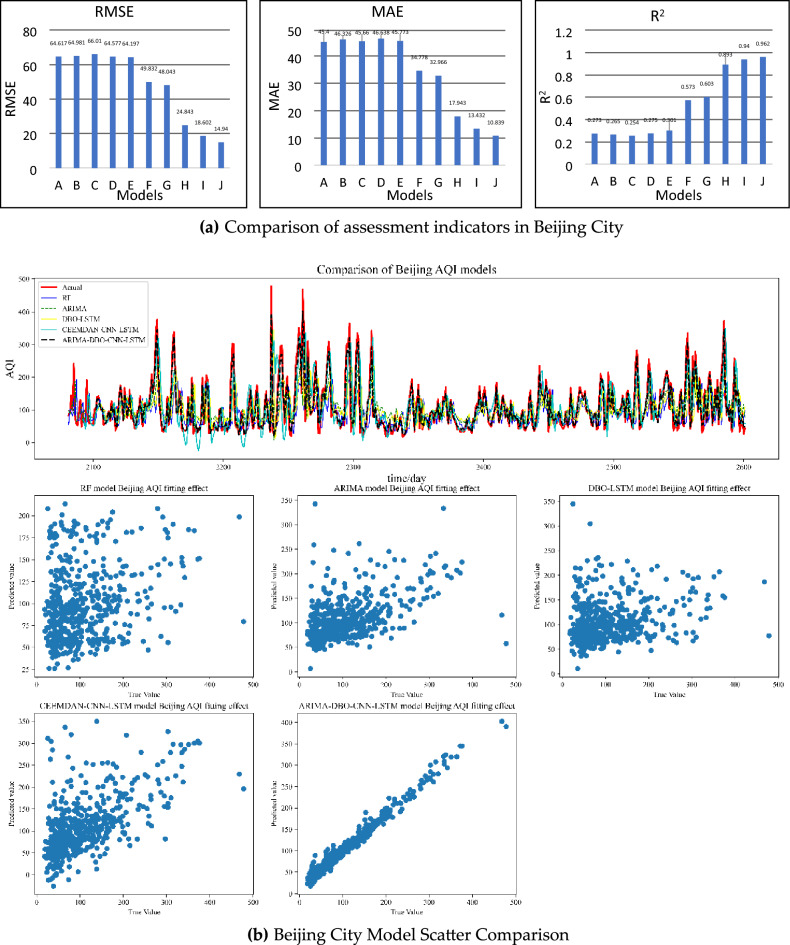
Beijing City Forecast Comparison.

**Figure 9:**
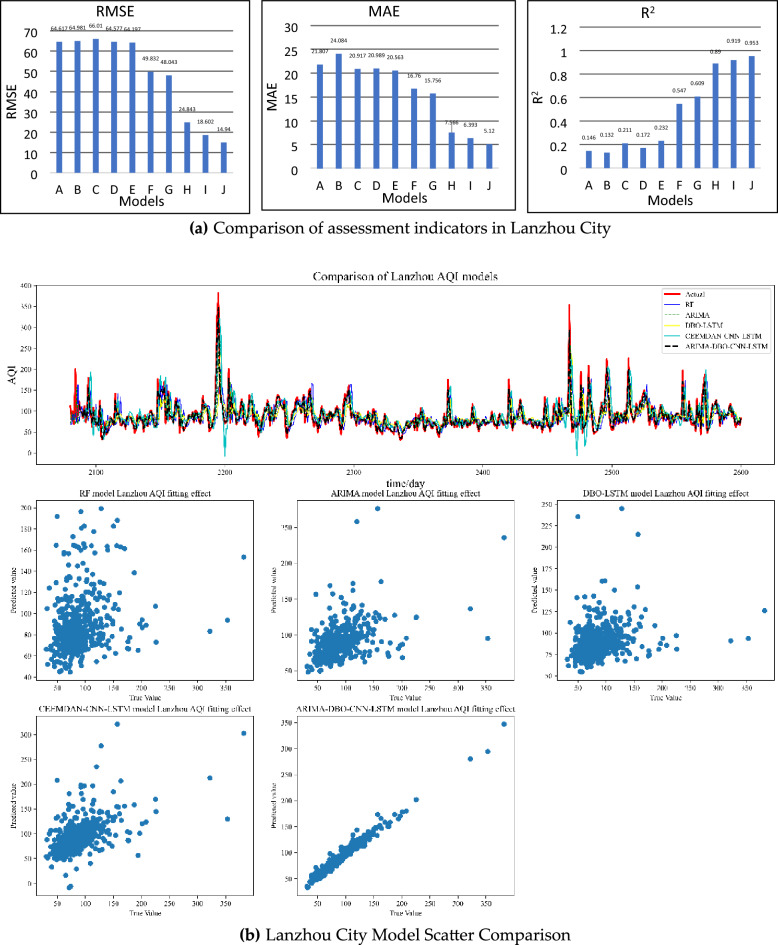
Lanzhou City Forecast Comparison.

**Figure 10:**
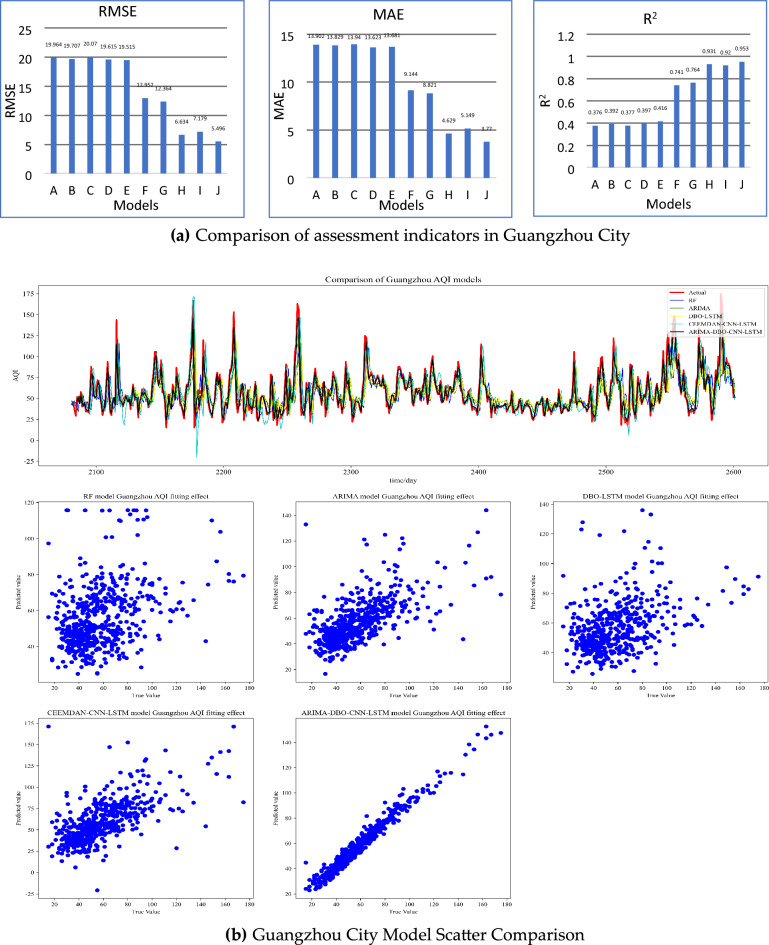
Guangzhou City Forecast Comparison.

Similar conclusions as Jiaozuo City can be drawn in the predictions of Beijing and Lanzhou, and the prediction pairs of Beijing and Lanzhou are shown in Figs. 8, 9 and 10. The combined prediction results of the three cities show that the ARIMA-DBO-CNN-LSTM model has the best prediction performance with an R^2^ index of 0.989.

## Discussion and conclusion

Air pollution is an environmental problem faced worldwide, and effective AQI prediction can help in air pollution management. Traditional time series prediction models have large prediction errors in air quality prediction, which increasingly cannot meet the needs of current production and life. However, neural networks represented by LSTM have shown excellent prediction performance in time series prediction. In this paper, we predict AQI of four cities by building ARIMA-DBO-CNN-LSTM models. To verify the advantages of the models, the comparison models are not limited to the selection of derived models, and the current mainstream algorithmic models for air quality prediction are incorporated.

We used the AQI data detected in four cities, Beijing, Lanzhou, Jiaozuo and Guangzhou, to construct and analyze all models, and the experimental results show that the ARIMA-DBO-CNN-LSTM has good prediction effect on the test set. The experimental results show that the model proposed in this paper outperforms the comparison models in terms of root mean square error (RMSE), mean absolute error (MAE) and coefficient of determination (R2). The RMSE values of the four cities are 7.594, 14.94, 7.841 and 5.496; the MAE values are 5.285, 10.839, 5.12 and 3.77; the R2 values are 0.989, 0.962, 0.953 and 0.953, respectively. The ARIMA-DBO-CNN-LSTM model has higher prediction accuracy for the four cities and better adaptability. Among the four selected Chinese cities, Jiaozuo city has the best prediction accuracy performance with three evaluation indexes of 7.594, 5.285 and 0.989 for RMSE, MAE and R2, respectively.

The model proposed in this paper also has the following problems: (1) The proposed model consists of a combination of two models, and each group of models can only fit part of the model better, but not 100%, which will produce reaveraging. (2) There are many external factors that affect the air quality (AQI) index, such as various meteorological indicators and seasonal factors, which are not considered in this paper.

In the future, various influencing factors can be introduced into the model to improve the accuracy of the model. In conclusion, this study shows that our proposed model can achieve higher accuracy than traditional single models such as BiLSTM, while the method based on EMD decomposition and LightGBM integration has better performance than other decomposition integration methods. In addition, the model is not complicated to construct and is worthy to be applied in practice.

## Data Availability

The data that support the findings of this study are available from [Resource and Environment Science and Data Center of the Chinese Academy of Sciences (https://www.resdc.cn/Default.aspx)] but restrictions apply to the availability of these data, which were used under license for the current study, and so are not publicly available. Data are however available from the authors upon reasonable request and with permission of [Resource and Environment Science and Data Center of the Chinese Academy of Sciences].
